# Syndrome hémolytique et urémique de l'enfant au Centre Hospitalier Universitaire (CHU) de Dakar: à propos de quatre observations

**DOI:** 10.11604/pamj.2016.24.138.8822

**Published:** 2016-06-10

**Authors:** Aliou Thiongane, Aliou Abdoulaye Ndongo, Idrissa Demba Ba, Djibril Boiro, Papa Moctar Faye, Younoussa Keita, Aïssatou Ba, Djeynaba Fafa Cissé, Idrissa Basse, Lamine Thiam, Indou Déme Ly, Babacar Niang, Abou Ba, Amadou Lamine Fall, Saliou Diouf, Ousmane Ndiaye, Mamadou Ba, Mamadou Sarr

**Affiliations:** 1 Centre Hospitalier National d'Enfants Albert Royer, Dakar, Sénégal; 2 Hôpital d'Enfants de Diamniadio, Dakar, Sénégal; 3 Service de Pédiatrie de l'Hôpital Abass Ndao, Dakar, Sénégal; 4 Service de Pédiatrie de l'Hôpital Aristide Le Dantec, Dakar, Sénégal

**Keywords:** Syndrome hémolytique et urémique, insuffisance rénale aiguë, thrombopénie, schizocytes, Hemolytic-uremic syndrome, acute renal failure, thrombopenia, schistocytes

## Abstract

Le syndrome hémolytique et urémique (SHU) est une cause fréquente d'insuffisance rénale aiguë (IRA) organique chez l'enfant. C'est une complication évolutive des gastroentérites aiguës (GEA) en particulier à Escherichia coli de l'enfant. Notre objectif était de décrire les aspects cliniques, thérapeutiques et évolutifs de cette affection chez quatre enfants. Nous avions colligé quatre cas de SHU. L’âge moyen était de 10,5 mois (5-15mois) exclusivement des garçons. L'examen clinique retrouvait une anémie de type hémolytique (pâleur et ictére), un syndrome oedémateux avec oligo-anurie (2 cas), une hypertension artérielle (1 patient), une GEA avec déshydratation sévère et choc hypovolémique (2 patients), des troubles de conscience. L'IRA était notée chez tous les patients de même que la thrombopénie et les schizocytes au frottis. Le Coombs direct était négatif. Il y avait une hyperkaliémie (3patients) dont 1 patient supérieure à 9,2 mmol/l, une hyponatrémie à 129mmol/l(1 patient) et une hypernatrémie à 153mmol/l (1 patient). Le shu était secondaire à une pneumonie à pneumocoque (1 patient), une GEA à E. coli (1 patient). Le traitement était essentiellement symptomatique et comprenait la restriction hydrique, la transfusion de concentrés érythrocytaires, les diurétiques, la dialyse péritonéale et l'hémodialyse. L’évolution était marquée par la survenue d'une insuffisance rénale chronique (1 patient) après 6 mois de suivi et la guérison (1 cas). Nous avions noté 3décés. Le SHU est la cause la plus fréquente d'IRA organique du nourrisson. Le diagnostic est essentiellement biologique, le traitement est surtout symptomatique.

## Abstract (en)

Hemolytic-uremic syndrome (HUS) is a common cause of organic acute renal failure (ARF) in children. It is a progressive complication of acute gastroenteritis (AGE), especially caused by Escherichia coli in children. This study aimed to describe the clinical, therapeutic and evolutionary aspects of this affection in four children. We collected four cases of HUS. The average age was 10,5 months (5-15mois), exclusively boys. Clinical examination revealed a hemolytic anemia (pallor and jaundice), oligoanuria and edematous syndrome (2 cases), arterial hypertension (1 patient), AGE associated with severe dehydration and hypovolemic shock (2 patients), consciousness disorders. ARF was found in all patients as well as thrombocytopenia and schizocytes smear. Direct Coombs test was negative. Hyperkalemia was found in 3 patients, of whom 1 with hyperkalemia level of more than 9.2 mmol/L, hyponatremia at 129 mmol/l (1 patient) and hypernatremia at 153 mmol/l (1 patient). HUS was secondary to pneumococcal pneumonia (1 patient) while AGE was secondary to E. coli (1 patient). The treatment was mainly symptomatic and included fluid restriction, transfusion of red cell concentrates, diuretics, peritoneal dialysis and hemodialysis. The evolution was marked by the onset of chronic renal failure (1 patient) after 6 months of follow-up and by recovery (1 case). Three patients died. HUS is the most common cause of organic acute renal failure in newborns. Diagnosis is essentially biological, treatment is mostly symptomatic.

## Introduction

Le syndrome hémolytique et urémique (SHU) est une cause fréquente d'insuffisance rénale chez l'enfant. Son incidence annuelle est estimée à 2,1 cas pour 100 000, avec un pic de 6,1 chez les enfants de moins de 5 ans [1–4]. C'est une complication évolutive non exceptionnelle des gastroentérites aiguës (GEA) de l'enfant, en particulier les GEA à Escherichia coli (E. coli). C'est pourquoi sa distribution suit la variation saisonnière des gastro-entérites à E.coli [4]. Il est responsable d'une morbidité importante et d'une mortalité non négligeable (10% de décès) à cause de l'insuffisance rénale aiguë qui le complique souvent [5]. Notre objectif était d’évaluer le devenir des patients atteints et de déterminer les complications de cette affection par une description des aspects cliniques, évolutifs, thérapeutiques et pronostiques chez quatre enfants.

## Méthodes

Il s'agissait d'une étude rétrospective de tous les cas de SHU colligés au Centre Hospitalier National d'Enfants Albert Royer de Dakar du 1^er^ Janvier 2009 au 30 Juin 2014 (5 ans et demi). Les données ont été recueillies à partir des dossiers des malades, des registres d'hospitalisation et du service d'information médicale. L'analyse a été faite avec le logiciel Epi info version 3.3.2.

## Résultats

**Observation 1:** garçon de 11 mois, sans antécédents médico-chirurgicaux particuliers qui était admis pour fiévre et difficultés respiratoires. L'examen clinique avait retrouvé une condensation pulmonaire droite, un souffle tubaire et une pâleur des muqueuses. La radiographie du thorax de face avait montré un foyer apical droit. La recherche d'antigènes solubles de pneumocoque dans les urines était positive. Devant ce Tableau 1 le diagnostic d'une pneumonie droite à pneumocoque était retenu et le traitement antibiotique avec de la pénicilline A démarré. Deux jours plus tard le patient avait présenté une aggravation de la pâleur cutanéo-muqueuse, un ictère franc, un syndrome œdèmateux, une anurie avec diurése inférieure à 0,5 ml/kg/h, une HTA systolique à 130/60 mmHg. L'examen des urines à la bandelette était normal. L'hémogramme avait montré une anémie hypochrome microcytaire avec un taux d'hémoglobine à 7,4g/dl, une thrombopénie sévére à 32000 éléments/mm^3^ et une légére hyperleucocytose à 12000 éléments/mm^3^ à prédominance neutrophile. Le frottis sanguin avait montré la présence de schizocytes avec 4 croix. Le Coombs direct était négatif. Il y avait une insuffisance rénale avec hyperazotémie à 2,92g/l (Normale 0,10-0,45) et une créatininémie à 40 mg/l (Normale inférieure à 13). L'ionogramme sanguin avait montré une hyponatrémie à 126 mEq/l (normale 135-145) et une hyperkaliémie sévére à 9,2 mEq/l (Normale 3,5-4,5). L’échographie abdominale avait montré la présence de gros reins hyperéchogènes avec conservation de la différenciation cortico-médullaire. L'examen anatomopathologique de la piéce de ponction biopsie rénale (PBR) avait montré des lésions de hyalinose segmentaire et focale touchant 50% des glomérules perméables, associées à des lésions de sclérose mésangiale diffuse, une néphrite interstitielle subaigüe modérée avec tubulopathie nécrotique probablement liée à l'infection pulmonaire. Devant ces éléments cliniques et paracliniques, le diagnostic de syndrome hémolytique et urémique atypique secondaire à une pneumonie à Streptococcus pneumoniae a été retenu. La prise en charge comprenait une transfusion de concentrés érythrocytaires et de PFC, une restriction hydrique, le traitement de l'hyperkaliémie et de l'HTA, une dialyse péritonéale puis une hémodialyse. L’évolution était partiellement favorable, avec survenue secondairement d'une insuffisance rénale chronique (IRC). Après six mois de suivi, l'enfant étaitréhospitalisé dans un tableau d'IRC terminale avec anémie, syndrome hémorragique, état d'anasarque avec polysérite, hypocalcémie. Le décès était survenu dans un tableau de sepsis grave avec coagulation intravasculaire disséminée (CIVD) une semaine après son admission.

**Tableau 1:** Résumé des caractéristiques cliniques et paracliniques des 4 observations

	Observation 1	Observation 2	Observation 3	Observation 4
Age (mois)	11	15	11	05
Sexe	Masculin	Masculin	Masculin	Masculin
Cas familiaux	-	-	-	-
phytothérapie	-	+	-	-
**SIGNES CLINIQUES**				
AEG	-	+	+	+
Pâleur	+	+	+	+
Ictère	+	-	-	-
Déshydratation	-	+	-	+
Diarrhée	-	+	-	+
Vomissements	-	+	-	+
Conscience	Claire	Altérée	Claire	Altérée
Diurèse	Anurie	Conservée	Non évaluée	Oligurie
Oedéme	+	-	+	+
HTA	+	-	-	-
**PARACLINIQUE**				
Anémie	+	+	+	+
Thrombopénie	+	+	+	+
Schizocytes	+	+	+	+
Insuffisance rénale	+	+	+	+
Ionogramme sanguin	Perturbé	Perturbé	Perturbé	Perturbé
Bactériologie	SP	E. Coli	Stérile	KP
PBR	+	-	-	-
**TRAITEMENT**				
Antibiotiques	+	+	+	+
Transfusion	CG	PFC	CG	-
Dialyse	+	-	+	-
Diurétiques	+	-	-	-
**EVOLUTION**	Décès	Guérison	Décès	Décès

AEG= altérationétatgénéral; HTA= hypertension artérielle; E. Col i= Escherichia coli; SP= Streptococcus pneumoniae; KP= Klebsiellapneumoniae; PBR= ponction-biopsierénale; CG= culotglobulaire; PFC= plasma fraiscongelé

**Observation 2:** garçon de 15 mois, qui était admis pour prise en charge d'une déshydratation sévère compliquée d'un état de choc hypovolémique secondaire à une gastroentérite aiguë. On notait également une pâleur et un subictére. Au plan paraclinique, il y avait une anémie hypochrome microcytaire avec un taux d'hémoglobine à 9,2 g/dl, des plaquettes à 111000 éléments/mm^3^ et une hyperleucocytose à 27000 éléments/mm^3^ à prédominance neutrophile. Le frottis sanguin avait montré la présence de schizocytes. Il y avait une insuffisance rénale aiguë avec azotémie à 0,89 g/l et créatininémie à 19,5mg/l. L'ionogramme sanguin avait montré une hypernatrémie à 154 mEq/l et une kaliémie à 3mEq/l. La coproculture avait identifié un E. coli. Le traitement était un apport hydrique en fonction de la diurèse. L’évolution était favorable avec guérison après deux semaines d'hospitalisation.

**Observation 3:** Garçon de 11 mois, cadet d'une fratrie de 9 enfants vivants et bien portants, hospitalisé pour prise en charge d'un syndrome œdémateux. Il n'y avait pas d'antécédents familiaux de SHU ni de cas similaire dans la famille. L'examen clinique avait retrouvé un syndrome d'anémie hémolytique associé à un syndrome œdémateux de type rénal avec une oligurie. Au plan paraclinique, on avait retrouvé une anémie normochromenormocytaire régénérative avec un taux d'hémoglobine à 6 g/dl, des plaquettes normales à 329000 éléments/mm^3^ et une hyperleucocytose à 13700 éléments/mm^3^ à prédominance neutrophile. Le frottis sanguin avait montré des schizocytes. Il y avait une IRA avec une azotémie à 1,42 g/l et une créatininémie à 41,64 mg/l. L'ionogramme sanguin avait montré une légère hyponatrémie à 129 mEq/l, une hyperkaliémie à 6,5 mEq/l. Par ailleurs il y avait une cytolyse hépatique avec ASAT à 317 UI/l et ALAT à 517 UI/l. Le traitement administré était une transfusion de concentré globulaire, une restriction hydrique et le traitement de l'hyperkaliémie. L’évolution était défavorable marquée par le décès au neuvième jour d'hospitalisation.

**Observation 4:** Garçon de 05 mois, unique de sa fratrie, issu d'un mariage consanguin au 3ème degré, qui avait été hospitalisé pour prise en charge d'une GEA évoluant depuis une semaine. Il n'y avait pas de notion de phytothérapie ni d'antécédents de SHU ou de cas similaires dans la famille. L'examen clinique avait trouvé un nourrisson en mauvais état général, avec une adynamie et une obnubilation avec un Glasgow pédiatrique à 13/15, une déshydratation sévère compliquée d'un état de choc, et un muguet. Au plan paraclinique on avait retrouvé une anémie normochromenormocytaire régénérative avec un taux d'hémoglobine à 5,3 g/dl, une thrombopénie sévère à 18000 éléments/mm^3^ et une hyperleucocytose à 13350 éléments/mm^3^ à prédominance neutrophile. Le frottis sanguin avait montré des schizocytes. La fonction rénale était altérée avec une azotémie à 0,69 g/l et une créatininémie à 12,19 mg/l. L'ionogramme sanguin avait montré une hypernatrémie à 153 mEq/l et une hyperkaliémie à 6,3mEq/l. La coproculture était négative. Le traitement a consisté en une antibiothérapie à base de ceftriaxone, associée à des transfusions de concentrés globulaires et de PFC. Le décès était survenu au 18^ème^ jour d'hospitalisation dans un tableau d'infection nosocomiale sévère à Klebsiella pneumoniae.

## Discussion

### Données épidémiologiques

*Incidence:* durant la période d’étude, du 1^er^ Janvier 2009 au 30 Juin 2014, 4720 patients ont été hospitalisés soit pour une gastro-entérite aigue (GEA) soit pour une pneumonie aigue à germe banal. Quatre observations ont été colligées soit une incidence de 0,1%. Au Sénégal, une étude menée entre 1998 et 1999 par Ndiaye O et al. [6] avait retrouvé une incidence plus élevée de 2,7% de SHU compliquant une GEA; tandis que Yameogo C. [7] avait retrouvé une incidence de 0,54% entre 1997 et 1999. Dans d'autres pays africains notamment au Maroc où l'analyse des données épidémiologiques d'une série à Rabat entre 2007 et 2011 avait révélé une incidence annuelle de 1,77/100 000 enfants de moins de 15 ans [8], tandisque la série de N. Nejjari et al de Casablanca [9] a montré une prévalence hospitalière de 0,2% entre 1990 et 1999. En Europe, notamment en France l'incidence annuelle était en moyenne de 1,8/100 000 enfants de moins de 5 ans et 0,7 chez les enfants < 15 ans entre 1993 et 1996 [10]. Entre 1996 et 2011 [11] elle était de 0,8/100 000 enfants de moins de 15 ans. Ces taux sont relativement élevés par rapport à ceux observés en Italie, où l′incidence est de 0,2/100 000 entre 1998 et 2000 [12]. Dans une étude prospective multicentrique menée en Allemagne et en Autriche entre 1997 et 2000, l'incidence du SHU était respectivement de 0.7/100 000 et 0.4/100 000 [13]. Nous notons que l'incidence du SHU est plus élevée dans les autres pays de l'Europe occidentale, notamment en Angleterre, où elle est de 1,54/100 000 chez les enfants de moins de cinq ans, alors qu′elle est de 3,4/100 000 en Ecosse et de 2,33/100 000 en Irlande [14]. Mais aussi, le même constat concerne le continent américain, où l'incidence rapportée aux Etats-Unis va de 0,2 à 3,4 [15].

*L’âge de survenue:* dans notre série l’âge moyen des patients était de 10,5 mois (5 - 15 mois) contrairement à ce qui a été décrit dans la plupart des études réalisées. Ainsi Ndiaye [6] au Sénégal avait retrouvé un âge moyen beaucoup plus élevé 33 mois. Au Maroc une étude rétrospective réalisée au CHU Ibn Rochd de Casablanca avait retrouvé un âge moyen de 16 mois [9] contre 37 mois dans lasérie de F.Bouziane de Rabat [8]. Par contre, en France, dans la série de L.A King, l’âge moyen était de 30 mois. Dans l’étude de R.Souza et al. réalisée au Brésil [16], portant sur 33 enfants l’âge moyen était de 2ans. C.Yameogo avait retrouvé un âge moyen de 53 mois [7]. La prédominance masculine a été notée dans toutes les études à l'exception de Ndiaye O. qui avait noté un sexe ratio de 1/2,5 [6]. Les données de la littérature et de notre série confirment que le SHU est une pathologie du nourrisson et du petit enfant, la moyenne d’âge variant de 10 mois à 2 ans selon les études avec une prédominance masculine.

*Distribution selon les saisons:* une tendance saisonnière a été retrouvée dans notre série avec un pic de fréquence au cours du 1er semestre de l'année (Janvier-Juin), ce qui est similaire aux données de la littérature en particulier ceux rapportés à partir de l’épidémie européenne de 2011 [11]. Ouzeddoun au Maroc [17], de même que Souza dans sa série réalisée au Brésil [16] ont retrouvé des résultats semblables. Une recrudescence durant la deuxième moitié de l'année (Juillet-Décembre) a été notée par C.Yameogo au Sénégal [7]. Ce qui explique l'augmentation de la fréquence du SHU pendant la période chaude et pluvieuse.

*Terrain:* dans notre série nous avions quatre cas de SHU: Les deux étaient des complications de GEA dont une à E.coli, ce sont des SHU typiques. Un cas était secondaire à une pneumonie à pneumocoque, c'est un SHU atypique. Pour le dernier cas, nous n'avons pas pu mettre en évidence la pathologie en cause. Aucun cas familial n'a été retrouvé. Ndiaye.O dans sa série avait retrouvé 100% de SHU typique [6]. Les résultats de cet auteur sont dus au fait qu'il n'avait pris en compte que les SHU secondaires à des gastro-entérites aigues. Au Zimbabwe, 37,5% des malades vivaient dans les milieux urbain et périurbain [18], tandis que dans notre série 50% résidaient dans ces milieux.

### Données cliniques et paracliniques

*Prodromes:* dans notre étude, les prodromes retrouvés sont sensiblement les mêmes que dans la plupart des travaux. La pâleur cutanéo-muqueuse est retrouvée chez tous nos patients, tandis que la diarrhée glaireuse n'a été rapportée que pour la moitié d'entre eux. Dans la plupart des travaux, l'intervalle entre le début de la diarrhée et la survenue de SHU est de 6 à 8 jours [7, 17, 19, 20], ce qui est semblable à nos constatations.

*Le syndrome hématologique:* sur le plan hématologique, l'anémie hémolytique avec présence de schizocytes a été retrouvée chez tous nos enfants. Nos résultats sont comparables aux données de la littérature. Le taux d'hémoglobine dans notre série était en moyenne de 6,97 g/dl. Nos chiffres se rapprochent des données des deux études antérieures Sénégalaises 7,37 g/dl [7], 5,9g/dl [6], Zimbabwéennes 6,1 g/dl [18] et européennes [12]. La thrombopénie est habituellement fréquente, elle a été notée chez 75% de nos patients, tandis que dans les autres études elle a atteint 85 à 93% [7, 15]. Contrairement à ce taux élevé de thrombopénie dans notre série, Ndiaye n'avait retrouvé que 29% de thrombopénie chez ses malades. L'absence de thrombopénie peut s'expliquer par la précocité de l'hémogramme puisque cette anomalie est plus due à une consommation excessive de plaquettes qu’à une production insuffisante [12, 20]. Nous avons par ailleurs noté une hyperleucocytose (moyenne de 15 000 /mm^3^) conformément à la série de Gerber et al. [13].

*Le syndrome rénal:* l'insuffisance rénale était constante. L'azotémie dans notre série était en moyenne de 1,48 g/l. Nos résultats sont comparables à ceux de la série de Ndiaye O (1,28 g /l) [6], de Souza R au Brésil (1,32 g /l) [16] et de Nathoo K J (2 g/l) [17]. La créatininémie était en moyenne de 28,33 mg/l, résultat assez proche de celui retrouvé par Gerber A de 25 mg/l [13]. D'autres études ont trouvé des chiffres supérieurs, c'est le cas du travail de Ndiaye au Sénégal avec un taux de 41,86 mg/l [6], de Yameogo avec 38 mg/l [7], et Souza au brésil avec 32,7mg/l [16]. L'insuffisance rénale retrouvée dans notre série était oligoanurique ou à diurèse conservée comme décrite dans la littérature [7, 9, 17]. L'hyponatrémie a été notée dans 50% des cas dans notre étude, Yameogo 88% [7]. L'hyperkaliémie menaçante (> 6 mEq/l) a été retrouvée chez 74% de nos patients motivant la mise en route d'un traitement hypokaliémiant et un monitoring continu de l'ECG. 25% de nos patients avaient présenté une HTA contre 61,5% [16].

*Les signes extra rénaux:* dans notre étude, 50% des patients présentaient des troubles neurologiques. Cette atteinte a été décrite dans la littérature avec des proportions allant de 17 à 52% [7, 8, 16, 17]. L'atteinte hépatique a été notée chez 50% de nos enfants, un peu moins dans la littérature 20 à 48% [9, 19].

*Facteurs étiologiques:* dans notre série, nous avons trouvé un cas de SHU typique compliquant une gastro-entérite à *E.coli*, et un cas de SHU atypique secondaire à une pneumonie à *Streptococcus pneumoniae*. Dans les études africaines, les germes les plus fréquemment retrouvés ont été *E.coli, Shigella dysenteria, Campylobacter jejuni et Streptococcus pneumoniae*.

*Données thérapeutiques:* le traitement symptomatique peut pallier aux conséquences les plus graves du SHU.

*Epuration extrarénale:* dans notre série deux patients ont été dialysés pour une durée allant de 1 semaine à 06 mois, contrairement à l’étude Sud-Africaine où la durée de la dialyse était de 1 à 17 jours [18]. Un seul patient a évolué vers l'IRC.

*La prise en charge de l'HTA:* dans notre série les inhibiteurs calciques ont été utilisés comme antihypertenseurs (1 patient), contrairement à la prise en charge de l'HTA décrite dans la littérature qui comprend l'utilisation des inhibiteurs de l'enzyme de conversion (IEC), des antagonistes des récepteurs de l'angiotensine II (ARAII), associés ou non aux bétabloquants et aux inhibiteurs calciques.

*Les antibiotiques:* étaient prescrits chez tous les patients, bien que leur utilisation doit être prudente car pouvant augmenter la lyse bactérienne massive avec libération de toxine [17].

*Traitement hématologique:* les transfusions des concentrés érythrocytaires ont été utilisées pour traiter les anémies sévères mal tolérées et les patients ayant présenté une thrombopénie sévère avec risque de saignement ont reçu une transfusion de plaquettes.

*Evolution et pronostic:* la mortalité dans notre série était très élevée (75%), même constat que pour Ndiaye et al (71%) [6]. Cependant, d'autres auteurs avaient trouvé des chiffres beaucoup plus faibles Yameogo (45%) [7] et Nathoo (40%) [18]. Ce taux élevé de mortalité était lié en partie à une insuffisance du plateau technique. Un seul cas d’évolution vers l'IRC a été noté chez nos patients, cet enfant est décédé 06 mois après le diagnostic. La PBR n'a été pratiquée que chez un malade présentant un SHU atypique secondaire à une infection invasive à pneumocoque. L'anurie est un facteur de mauvais pronostic surtout si sa durée est supérieure à 7 jours d'après les données de la littérature. Ainsi, il existe une relation étroite entre la durée de l'anurie et celle des dialyses avec un retentissement péjoratif sur la survie des enfants. Il existerait une corrélation entre la présence de signes extra-rénaux sévères et une évolution péjorative.

## Conclusion

Le SHU est la cause la plus fréquente d'IRA chez le nourrisson. C'est une complication des gastroentérites à E. Coli et des pneumonies à pneumocoque. Le diagnostic est essentiellement biologique, le traitement est surtout symptomatique.

### Etat des connaissances sur le sujet

- Le SHU de l'enfant existe sous deux formes: typique et atypique;
- L’épuration extra-rénale permet d'améliorer le pronostic.

### Contribution de notre étude a la connaissance

- La morbi-mortalité est élevée;
- Etat des lieux sur la prise en charge du SHU au Sénégal.

## References

[1] Hill Gs (1996). La Biopsie Rénale Dans Les Syndromes Hémolytiques Et Urémiques. La Biopsie Rénale..

[2] Loirat C (2012). Syndrome Hémolytique Et Urémique Chez L'enfant. Emc-Pédiatrie..

[3] Loirat C (2001). Syndrome Hémolytique Et Urémique Typique Post-Diarrhée: Aspects Cliniques. Arch Pediatr..

[4] Gasser C, Gauthier E, Steck A (1955). Hemolytic-Uremic Syndrome: Bilateral Necrosis Of The Renal Cortex In Acute Acquired Hemolytic Anemia. Schweiz Med Wochenschr..

[5] Karmali Ma, Petric M, Lim C (1985). The Association Between Idiopathic Hemolytic Uremic Syndrome And Infection By Vérotoxine-Producing Escherichia Coli. J Infect Dis..

[6] Ndiaye O, Sylla A, Diagne I (2001). Le Syndrome Hémolytique Et Urémique: Une Complication Des Gastroentérites Aigues De L'enfant. Dakar Médical..

[7] Yameogo C (2000). Syndrome Hémolytique Et Urémique Chez L'enfant: à Propos de 42 cas Colligés A L'hôpital Principal De Dakar. Thèse Médecine.

[8] Bouziane F (2011). Syndrome Hémolytique Et Urémique Chez L'enfant: à Propos De 15 Cas. Thèse Médecine.

[9] Nejjari N, Ait Sab I, Bouharrou A (2004). Le Syndrome Hémolytique Et Urémique: à Propos De 23 Cas. Mag Med..

[10] Decludt B, Bouvet P, Mariani-Kurkdjian P (2000). Haemolytic Uraemic Syndrome And Shiga Toxin-Producing Escherichia Coli Infection In Children In France. Epidemiol Infect..

[11] King LA, Mariani-Kurkdjian P, Gouali M (2013). Surveillance Du Syndrome Hémolytique Et Urémique Chez Les Enfants De Moins De 15 Ans En France, 1996-20. Arch De Pédiatrie..

[12] Tozzi AE, Caprioli A, Minelli F (2003). Shiga toxin-producing Escherichia coli infections associated with hemolytic uremic syndrome, Italy, 1988-2000. Emerg Infect Dis..

[13] Gerber A, Karch H, Allerberger F (2002). Clinical Course And The Role Of Shiga Toxin-Producing Escherichia Coli Infection In The Hemolytic-Uremic Syndrome In Pediatric Patients, 1997- 2000, In Germany And Austria: A Prospective Study. J Infect Dis..

[14] Lynn RM, O'brien SJ, Taylor CM (2005). Childhood Hemolytic Uremic Syndrome, United Kingdom And Ireland. Emerg Infect Dis..

[15] Haeghebaert S, Vaillant V, Espie E (2001). Surveillance Du Syndrome Hémolytique Et Urémique Chez Les Enfants De Moins De 15 Ans En France. BEH..

[16] Souza R, Carvalhaes JA, Nishimura L (2011). Hemolytic Uremic Syndrome In Pediatric Intensive Care Units In São Paulo, Brazil. Open Microbiol J..

[17] Ouzeddoun N, Rhou H, Benamar L (1997). Le syndrome hémolytique et urémique: à propos de 5 cas. MAG..

[18] Nathoo KJ, Sanders JA, Siziya S, Mucheche C (1995). Haemolytic Uaremic Syndrome Following Shigella Dysenteriae Type 1 Outbreak In Zimbabwe: A Clinical Experience. Cent Afr J Med..

[19] Bernard A, Tounian P, Leroy B (1996). Les Manifestations Digestives Du Syndrome Hémolytique Et Urémique De L'enfant. Arch Pédiatr..

[20] Cochran B, Valerie M, Frederic W (2004). Pneumococcus-Induced T-Antigen Activation In Hemolytic Uremic Syndrome And Anemia. Pediatr Nephrol..

